# Rate and Determinants of Excessive Fat-Free Mass Loss After Bariatric Surgery

**DOI:** 10.1007/s11695-020-04654-6

**Published:** 2020-05-15

**Authors:** Malou A. H. Nuijten, Valerie M. Monpellier, Thijs M. H. Eijsvogels, Ignace M. C. Janssen, Eric J. Hazebroek, Maria T. E. Hopman

**Affiliations:** 1 Radboud Institute for Health Sciences, Department of Physiology (392), Radboud University Medical Center, P.O. Box 1901, 6500 HB Nijmegen, The Netherlands; 2 Nederlandse Obesitas Kliniek, Huis ter Heide, The Netherlands; 3 Department of Surgery, Rijnstate Hospital/Vitalys Clinics, Arnhem, The Netherlands

**Keywords:** Bariatric surgery, Fat-free mass, Body composition

## Abstract

**Purpose:**

Fat-free mass (FFM) loss is a concerning aspect of bariatric surgery, but little is known about its time-course and factors related with excessive FFM loss. This study examined (i) the progress of FFM loss up to 3 years post-bariatric surgery and (ii) the prevalence and determinants of excessive FFM loss.

**Materials and Methods:**

A total of 3596 patients (20% males, 43.5 ± 11.1 years old, BMI = 44.2 ± 5.5 kg/m^2^) underwent sleeve gastrectomy (SG) or Roux-en-Y gastric bypass (RYGB) surgery. Bioelectrical impedance analysis was performed preoperatively and 3, 6, 9, 12, 18, 24 and 36 months post-surgery. Changes in body composition were assessed by mixed model analysis. Prevalence of excessive FFM loss (based on three different cutoff values: ≥ 25%, ≥ 30% and ≥ 35% FFM loss/weight loss (= %FFML/WL)) was estimated and its determinants were assessed by linear regression analysis.

**Results:**

Highest rates of FFM loss were found at 3 and 6 months post-surgery, reflecting 57% and 73% of peak FFM loss, respectively. Prevalence of excessive FFM loss ranged from 14 to 46% at 36 months post-surgery, with an older age (*β* = 0.14, 95%CI = 0.10–0.18, *P* < .001), being male (*β* = 3.99, 95%CI = 2.86–5.12, *P* < .001), higher BMI (*β* = 0.13, 95%CI = 0.05–0.20, *P* = .002) and SG (*β* = 2.56, 95%CI = 1.36–3.76, *P* < .001) as determinants for a greater %FFML/WL.

**Conclusion:**

Patients lost most FFM within 3 to 6 months post-surgery. Prevalence of excessive FFM loss was high, emphasizing the need for more vigorous approaches to counteract FFM loss. Furthermore, future studies should assess habitual physical activity and dietary intake shortly after surgery in relation to FFM loss.

**Electronic supplementary material:**

The online version of this article (10.1007/s11695-020-04654-6) contains supplementary material, which is available to authorized users.

## Introduction

Bariatric surgery is considered the most effective strategy in patients with morbid obesity to achieve long-lasting weight loss, improve quality of life and reduce comorbidities [1, 2]. However, bariatric surgery is also associated with nutritional deficiencies and excessive loss of fat-free mass (FFM) [3, 4]. Since FFM consists for 30–50% of muscle mass, it plays an important role in several metabolic mechanisms, such as functional capacity, resting energy expenditure, thermoregulation and bone (re)modelling [5]. Therefore, excessive FFM loss is detrimental for patients because this may lead to difficulties in daily life activities, lower quality of life, weight regain, fat accumulation and higher risk of developing sarcopenia and osteoporosis [5–8]. These consequences are especially of concern in post-bariatric patients since they may counteract the long-term success of surgery in terms of weight loss, quality of life and reduction of comorbidities.

Previous studies reported average FFM reductions of 3–14 kg within 1 year post-surgery, whereas large interindividual variations in FFM loss were observed across patients [9–11]. These observations suggest that post-bariatric patients can already lose a substantial amount of FFM within 1 year post-surgery. However, little is known about the time-course of excessive FFM loss, since longitudinal studies with repetitive FFM measurements are scarce due to the radiation exposure of repetitive DEXA measurements. Furthermore, insight into the prevalence and determinants of excessive FFM loss could help to identify bariatric patients at risk.

We examined (i) the progress of FFM loss up to 3 years post-bariatric surgery, (ii) the prevalence of excessive fat-free mass loss and (iii) determinants of excessive FFM loss. For this purpose, we analysed changes in body composition following bariatric surgery, using a large Dutch bariatric population. We hypothesized that FFM is predominantly lost within 6 months post-surgery, due to the acute impact of bariatric surgery on dietary intake. The prevalence of excessive FFM loss is expected to be substantial, whereas the magnitude of FFM loss may be associated with factors such as age, sex, preoperative BMI and type of surgery, because of the age-related decline in muscle protein synthesis and functional differences between bariatric procedures.

## Materials and Methods

### Study Population

In this retrospective study, data was extracted from the electronic medical reports of the Nederlandse Obesitas Kliniek (NOK, Dutch Obesity Clinic), a national clinic providing an extensive perioperative care programme for bariatric patients [12]. The NOK screens their patients on eligibility for bariatric surgery, based on the IFSO guidelines [13], including (i) BMI > 40 kg/m^2^ or BMI > 35 kg/m^2^ with comorbid conditions, (ii) > 6-month serious weight loss attempts and (iii) no psychological dysfunction with increased risk on causing medical problems. For the present study, all patients who underwent a primary laparoscopic Roux-en-Y gastric bypass (RYGB) surgery or sleeve gastrectomy (SG) between January 2015 and April 2016 were included. Patients who underwent a revisional bariatric procedure were excluded.

### Perioperative Care Programme

The content of the NOK perioperative care programme was previously described in detail [12]. In short, the NOK provides an interdisciplinary care programme for bariatric patients consisting of pre- and post-bariatric group counseling focused on education about lifestyle change. The 7-week preoperative programme consists of weekly group visits containing three 1-h sessions with a dietician, psychologist and physiotherapist, respectively. After the preoperative programme, the bariatric procedure is performed, followed by a 15-month postoperative care programme. Again, patients visit the clinic once every 3 to 9 weeks for (group) sessions with a dietician, physiotherapist and psychologist with the aim to adopt a healthy lifestyle. During the perioperative care programme, the patient’s progress is monitored with regular assessment of weight and body composition up to 5 years post-surgery. Patients have regular follow-ups with a physician (at 3 weeks and 3, 6, 9, 12 and 18 months after surgery). During these medical checks both  weight and FFM loss are assessed by the bariatric care team. When FFM loss is deemed extensive by the treating physician, reasons for the extensive loss are assessed and, if necessary, treated by the physician. Moreover, patients will have extra individual consultations with the physician and/or dietician until FFM loss is halted. Nevertheless, there is currently no standardized protocol for the treatment of FFM loss.

### Data Collection

All data was collected by trained personnel of the NOK and directly uploaded into the patient’s electronic medical record, which automatically detects errors or incorrect data to minimize human errors. At the start of the preoperative care programme, patient characteristics are collected, including age and sex. Furthermore, presence of obesity-related comorbidities such as hypertension, sleep apnoea, dyslipidaemia, arthrosis and diabetes mellitus was assessed by a physician based on information of the referring physician.

### Weight and Body Composition

Weight and body composition measures were assessed preoperatively, and at 3, 6, 9, 12, 18, 24 and 36 months post-surgery. Height and waist circumference were measured using a non-elastic measuring tape. Body weight, fat percentage, fat mass and FFM were determined by bioelectrical impedance analysis (TANITA® brand, model BC-420MA) [14]. Percentages of total weight loss (%TWL), excess weight loss (%EWL), fat mass loss and FFM loss with respect to preoperative measures were calculated for each postoperative time point. Furthermore, the proportion of FFM loss from total weight loss (expressed in %FFML/WL) was calculated at each follow-up point as follows:

$$ \%\mathrm{FFML}/\mathrm{WL}=\frac{\mathrm{FFM}\left(\mathrm{post}\right)-\mathrm{FFM}\left(\mathrm{preoperative}\right)}{\mathrm{Weight}\left(\mathrm{post}\right)-\mathrm{Weight}\left(\mathrm{preoperative}\right)}\times 100\% $$

Currently, no guidelines are available that define how much FFM loss is excessive after bariatric surgery. According to the Quarter FFM Rule, in healthy weight loss, the proportion of weight loss that can be attributed to FFM is around 25% [15]. Nevertheless, former studies in post-bariatric populations have showed that FFM loss after bariatric procedures, such as RYGB, is expected to be greater than 25% [9, 16]. In this study, we used three different cutoff values to determine presence of excessive FFM loss: 25%, 30% and 35% FFM loss of total weight loss (=FFML/WL). At each follow-up point, patients were allocated to the proportional or the excessive loss group, based on each cutoff value (≥ 25%-, ≥ 30%- and ≥ 35%FFML/WL, respectively).

### Statistical Analysis

Statistical analyses were performed using SPSS (IBM SPSS Statistics for Windows, Version 24 IBM Corp., Armonk, NY, USA.). All continuous variables were visually inspected and tested for normality by the Shapiro-Wilk test, to decide for either parametric or non-parametric statistical analyses. Changes in body composition parameters up to 36 months post-surgery were assessed using mixed model analyses. To examine determinants of FFM loss, univariate and multivariate linear regression was performed with both 12-month and 24-month %FFML/WL as dependent variable and age, sex, type of surgery, preoperative BMI, and comorbidities as covariates. Moreover, a univariate and multivariate logistic regression analysis was performed on 12-month and 24-month excessive FFM loss (defined as ≥ 25%FFML/WL) with the same covariates. To assess the effect of missing data, analyses were performed for the total cohort (all patients) and a subgroup of patients with maximum 1 missing value between the preoperative and 36-month measurement (full data analysis). Statistical significance was assumed at *P* < .05 (two-sided).

## Results

The total cohort consisted of 3596 patients (80% females) with an age of 43.5 ± 11.1 years and a preoperative BMI of 44.2 ± 5.5 kg/m^2^. A total of 3022 patients (84%) underwent a RYGB, whereas 574 patients (16%) underwent a SG. Preoperative prevalence of comorbidities was 1324 patients (36.8%) with hypertension, 710 patients (19.7%) with dyslipidaemia, 467 patients (13.0%) with sleep apnoea, 437 patients (12.2%) with arthrosis and 783 patients (21.8%) with diabetes mellitus. Preoperative body composition parameters are summarized in Table 1.

**Table 1:** Changes in body composition parameters up to 36 months post-surgery

	Preoperatively (*n* = 3596)	3 months (*n* = 3316)	6 months (*n* = 3421)	9 months (*n* = 3417)	12 months (*n* = 3334)	18 months (*n* = 2335)	24 months (*n* = 2715)	36 months (*n* = 1193)	Time *P* value^a^
BMI, kg/m^2^	44.2 ± 5.5	36.1 ± 5.1*	33.0 ± 4.9*	30.9 ± 4.8*	30.0 ± 4.8*	29.4 ± 4.8*	29.8 ± 5.0*	31.0 ± 5.3*	< .001
Weight, kg	127.5 ± 20.4	104.1 ± 17.8*	95.3 ± 17.0*	89.1 ± 16.6*	86.7 ± 16.4*	84.9 ± 16.6*	85.7 ± 17.0*	89.2 ± 18.5*	< .001
FM, kg	62.9 ± 13.5	45.0 ± 12.5*	37.7 ± 11.7*	32.7 ± 11.5*	31.1 ± 11.3*	30.0 ± 11.1*	30.9 ± 11.5*	34.0 ± 12.2*	< .001
%FM	49.3 ± 5.5	42.8 ± 7.2*	39.1 ± 7.9*	36.0 ± 8.3*	35.1 ± 8.3*	34.8 ± 8.4	35.0 ± 9.4	37.4 ± 7.9*	< .001
FFM, kg	64.3 ± 11.5	59.0 ± 10.9*	57.5 ± 10.8*	56.4 ± 10.8*	55.6 ± 10.7*	54.8 ± 10.5*	54.8 ± 10.4	55.2 ± 10.8	< .001
AC, cm	129.5 ± 13.6	111.5 ± 13.1*	104.7 ± 20.5*	100.7 ± 30.4*	98.1 ± 20.8*	97.8 ± 39.8	99.7 ± 48.0*	101.6 ± 30.0	< .001
%TWL	NA	18.3 ± 4.1	25.4 ± 5.2*	30.1 ± 6.6*	31.9 ± 7.3*	33.1 ± 8.4*	32.2 ± 9.1*	29.9 ± 9.5*	< .001
%EWL	NA	44.4 ± 12.9	61.0 ± 16.3*	72.3 ± 19.1*	76.5 ± 20.4*	79.4 ± 22.0*	77.2 ± 23.0*	70.9 ± 23.2*	< .001

Values are displayed as mean ± SD. *BMI* body mass index, *FM* fat mass, *%FM* fat percentage, *FFM* fat-free mass, *AC* abdominal circumference, *%TWL* percentage of total weight loss, *%EWL* percentage of excess weight loss, *NA *not applicable

^a^Overall *P* value for mixed model on body composition over time

*P < .05 with respect to former measurement

### Changes in Body Composition over Time

Body composition parameters were significantly lower at each follow-up measurement compared with preoperative measures (all *P* < .001; Table 1). Most favourable body composition, i.e. lowest weight, BMI and fat percentage, was reached at 18 months post-surgery with a corresponding 33.1% TWL and 79.4% EWL. After this 18-month time point, some weight regain of approximately 4.3 kg occurred up to 36 months post-surgery, which mainly consisted of a significant increase in fat mass (+ 4.0 kg) whereas FFM stabilized after 18 months post-surgery.

**Fig. 1:**
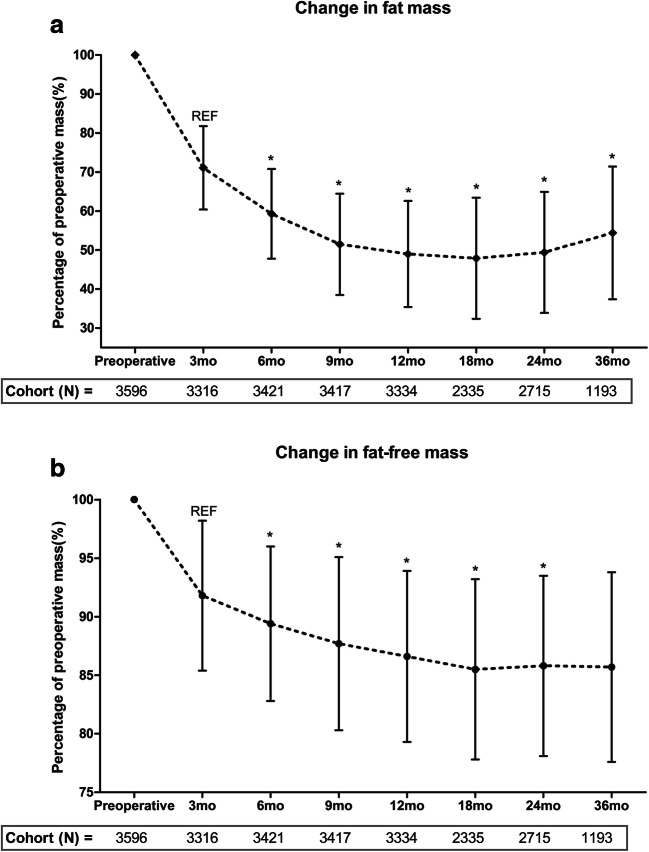
Changes in fat mass (**a**) and fat-free mass (**b**) with respect to preoperative measures up to 36 months post-surgery. Error bars reflect standard deviation (1SD). *P < 0.05 with respect to former measurement. Fat mass significantly decreased to 52.1% of preoperative fat mass at 18 months post-surgery, followed by a significant increase in fat mass. FFM significantly decreased to 14.5% of preoperative FFM at 18 months post-surgery, with no significant changes up to 36 months post-surgery. Highest rates of fat mass loss and FFM loss were observed at 3 and 6 months post-surgery

Post-surgery changes of fat mass loss and FFM loss are displayed in Fig. 1. Lowest fat mass and FFM were both reached at 18 months post-surgery, with 52.1 ± 15.5% loss of preoperative fat mass and 14.5 ± 7.7% loss of preoperative FFM. Fat mass was significantly lower compared with each former measurement up to 18 months post-surgery, and subsequently increased up to 36 months post-surgery (Fig. 1a). Likewise, FFM was significantly lower compared with each former time point up to 18 months post-surgery, but no significant changes occurred up to 36 months post-surgery (Fig. 1b). We also observed a large interindividual variability of FFM loss, with a 95%CI from − 1.02 to 29.97% of preoperative FFM at 18 months post-surgery.

The highest rate of FFM loss occurred in the first 6 months after surgery, with already 57% FFM loss of the peak FFM loss (i.e. 18-month FFM loss) after 3 months and 73% FFM loss after 6 months. The same pattern was seen for fat mass loss, with 55% and 78% loss of 18-month peak fat mass loss for 3 months and 6 months, respectively. Full data analyses of the group with maximum 1 missing value showed similar patterns of fat mass loss (18-month fat mass loss 50.4 ± 17.9%, with 54% and 77% of the peak loss at 3 and 6 months, respectively) and FFM loss (18-month FFM loss 15.1 ± 7.3%, with 55% and 74% of peak loss at 3 and 6 months, respectively) (see Supplemental Figure 1).

### Prevalence of Excessive FFM Loss

**Fig. 2:**
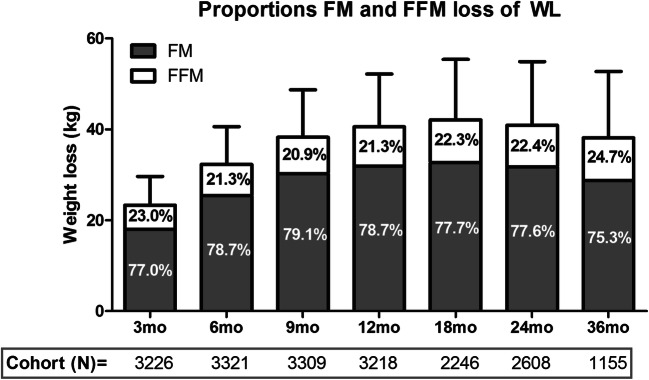
Weight loss with respect to preoperative weight with its proportions of fat mass loss and FFM loss. Bars reflect weight loss in kilogrammes with standard deviation. Percentages of fat mass loss and FFM loss are displayed within the bars. FM, fat mass; FFM, fat-free mass. Proportion of FFM loss of total weight loss decreased from 3 to 9 months post-surgery and subsequently increased again up to 24.7% at 36 months post-surgery

Proportions of fat mass loss and FFM loss within total weight loss are displayed in Fig. 2. At 3 months post-surgery, patients lost on average 5.4 ± 4.7 kg FFM with respect to 23.3 ± 6.3 kg of weight, reflecting a proportion of 23.0%. This proportion of FFM loss decreased to 20.9% at 9 months post-surgery and subsequently increased again to 24.7% up to 36 months post-surgery.

**Fig. 3:**
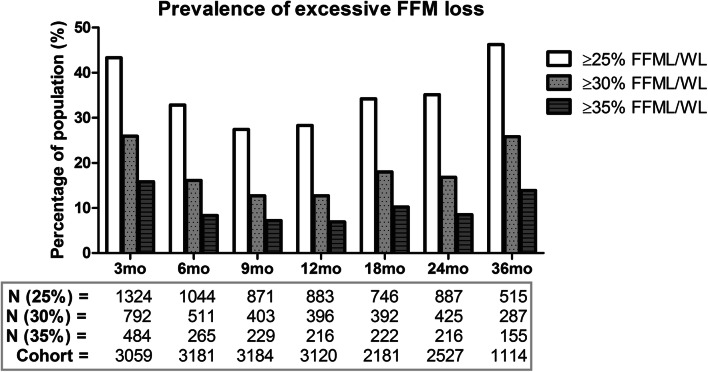
Prevalence of excessive FFM loss in our cohort at each measuring point based on the cutoff values of ≥ 25%, ≥ 30% and ≥ 35% FFML/WL. For each cutoff value, prevalence of excessive FFM loss decreased from 3 to 9 months post-surgery. Thereafter, prevalence increased again up to 36 months post-surgery

Based on the cutoff value of 25%, excessive FFM loss was found in 1324 patients (43.3%) at 3 months post-surgery (Fig. 3). This prevalence of excessive FFM loss decreased to 28.3% at 12 months post-surgery, followed by an increase in prevalence up to 46.2% at 36 months post-surgery. Prevalences of excessive FFM loss based on the cutoff values of 30% and 35% showed a similar pattern over time, with lower prevalences ranging from 12.7 to 25.9% and from 6.9 to 15.8% for the 30% and 35% cutoff values, respectively.

### Determinants of FFM Loss

Univariate linear regression analysis revealed a significant impact of all covariates on %FFML/WL at 12 months (Table 2). Even greater effect sizes were found for the association of covariates on %FFML/WL at 24 months. An older age, being male, SG, having a higher preoperative BMI, and presence of hypertension, dyslipidaemia, arthrosis or diabetes were related to a greater %FFML/WL. All covariates were subsequently included in the multivariate model. This multivariate model showed similar results with older age, male gender, SG and higher preoperative BMI as determinants for a greater %FFML/WL both at 12 months and 24 months post-surgery. Nevertheless, the significant associations of %FFML/WL with preoperative comorbidities disappeared in the multivariate model, with the exception of dyslipidaemia at 24 months post-surgery. Overall fit (i.e. *R*^2^) of the multivariate model was 3.4% for 12-month %FFML/WL and 5.0% for 24-month %FFML/WL. Logistic regression on 12-month and 24-month excessive vs. non-excessive FFM loss (based on cutoff value of 25%FFML/WL) confirmed the results of our linear model: older age, male gender, higher preoperative BMI and SG are related to excessive FFM loss (Supplemental table 1).

**Table 2:** Univariate and multivariate linear regressions on 12-month and 24-month %FFML/WL

	12 months (*n* = 3119)			24 months (*n* = 2526)		
	Beta-coefficient	95%CI	*P*	Beta-coefficient	95%CI	*P*
Univariate						
Age	0.077	0.047 to 0.108	*< .001*	0.140	0.101 to 0.179	*< .001*
Sex (ref = male)	− 2.689	− 3.579 to − 1.800	*< .001*	− 3.986	− 5.116 to − 2.855	*< .001*
Type of surgery (ref = RYGB)	2.540	1.603 to 3.476	*< .001*	2.561	1.360 to 3.763	*< .001*
Preop BMI	0.091	0.028 to 0.154	*.005*	0.124	0.045 to 0.203	*.002*
Hypertension	1.540	0.844 to 2.236	*< .001*	2.480	1.322 to 3.338	*< .001*
Dyslipidaemia	1.752	0.900 to 2.605	*< .001*	3.113	2.079 to 4.146	*< .001*
Sleep apnoea	1.664	0.649 to 2.678	*.001*	1.259	0.002 to 2.517	.05
Arthrosis	1.328	0.293 to 2.363	*.012*	1.489	0.245 to 2.733	*.019*
Diabetes	1.664	0.840 to 2.488	*< .001*	2.507	1.495 to 3.518	*< .001*
Multivariate						
Age	0.056	0.019 to 0.093	*.003*	0.116	0.069 to 0.163	*< .001*
Sex (ref = male)	− 2.096	− 3.022 to − 1.171	*< .001*	− 3.273	− 4.435 to − 2.111	*< .001*
Type of surgery (ref = RYGB)	2.593	1.630 to 3.555	*< .001*	2.896	1.670 to 4.122	*< .001*
Preop BMI	0.100	0.035 to 0.164	*.002*	0.153	0.073 to 0.232	*< .001*
Hypertension (ref = no)	0.450	− 0.360 to 1.259	.28	0.579	− 0.412 to 1.569	.25
Dyslipidaemia (ref=no)	0.605	− 0.382 to 1.592	.23	1.526	0.336 to 2.716	*.012*
Sleep apnoea (ref=no)	0.541	− 0.505 to 1.587	.31	− 0.513	− 1.791 to 0.764	.43
Arthrosis (ref=no)	0.729	− 0.336 to 1.794	.18	0.376	− 0.891 to 1.643	.56
Diabetes (ref=no)	0.616	− 0.336 to 1.567	.21	0.651	− 0.509 to 1.812	.27

Overall fit of multivariate model was *R*^2^ = 0.034 for 12 months post-surgery and *R*^2^ = 0.050 for 24 months post-surgery

## Discussion

The present study examined the progress of FFM loss up to 36 months after bariatric surgery and determined the prevalence and determinants of excessive FFM loss. We found that patients lose a substantial amount of FFM after bariatric surgery, with the highest rate at 3 to 6 months post-surgery. Furthermore, prevalence of excessive FFM loss ranged from 14 to 46% at 36 months post-surgery, dependent on the cutoff value, with an older age, being male, higher preoperative BMI and SG as determinants of a greater proportion of FFM loss. These findings indicate that FFM loss is a substantial problem which occurs in a large part of the post-bariatric patients, while counteracting measures should be applied within 3 to 6 months post-surgery.

Our study shows that FFM loss seems excessive in at least 1 out of 7 patients (i.e. 14% of the patients exceeds the 35%FFML/WL threshold at 36 months post-surgery). Although no previous studies assessed prevalence of excessive FFM loss, substantial amounts of FFM loss with large variation between individuals have been reported in other studies [17–19]. These findings suggest that FFM loss is not sufficiently tackled by post-bariatric care. Because of the essential role of FFM in several processes (e.g. metabolic health, functional capacity and bone remodelling), excessive FFM loss could potentially lead to decreases in resting energy expenditure, loss of bone mass, difficulties in daily life activities and demotivation for exercise [5, 20, 21]. These consequences could eventually counteract the success of bariatric surgery and increase health risks.

This is the first study to longitudinally assess FFM loss following bariatric surgery with repetitive measurements in the first year post-bariatric surgery as well as a long-term follow-up. We found that the highest rates of FFM loss were observed at 3 and 6 months post-surgery, and a plateau phase was reached at 18 months post-surgery. Our findings align with previous studies reporting substantial FFM loss at 6 months post-surgery with little change up to 12 months post-surgery [17, 18].

We also found that being male, older ager, sleeve gastrectomy and higher preoperative BMI were related to greater FFM loss. These determinants of FFM loss could help to identify patients at risk. A previous study also identifies high preoperative BMI and being male as determinants of post-bariatric FFM loss [11]. A potential explanation for these findings may relate to the larger preoperative FFM and differences in muscle fibre composition between men and women. Large muscles predominantly consist of type II fibres, which are more susceptible to atrophy in periods of disuse or decreased energy intake [22]. Female skeletal muscle also has a higher ability to metabolize fat lipids and, therefore, better adapt to nutrient deprivation [23]. Furthermore, the effect of age on FFM loss could be explained by the age-related decline in muscle mass, which accelerates with higher age. Therefore, older patients inherently have a diminished muscle protein synthetic response to anabolic stimuli [24] and are already more prone to lose FFM compared with younger patients.

One unexpected finding of the present study was the higher FFM loss in SG compared with RYGB surgery. A potential explanation for our findings may relate to confounding by indication. Our SG patients had a higher preoperative BMI (47.0 ± 7.4 kg/m^2^ vs. 43.7 ± 4.9) and were relatively older (44 ± 11 vs. 39 ± 12 years) and more often male (24% vs. 19%) compared with RYGB patients. These patient characteristics were already related to a higher FFM loss and could therefore have distorted our findings on type of surgery.

Our prediction model only explained 3.4 to 5.0% of the variation in FFM loss, suggesting a multifactorial process in which other factors are of greater importance. Especially physical activity and dietary intake are known to have an essential role in muscle synthesis and breakdown [5]. Although all patients were actively coached on these factors by our care team, no information on exact nutrient intake and exercise patterns of our population can be given since they were not assessed during the programme. Current literature also lacks regular, concurrent and objective assessment of physical activity patterns and dietary intake within 6 months post-bariatric surgery, which emphasizes the need for such studies to understand the role of these factors in FFM loss.

This is the first study assessing FFM loss in a large nationwide cohort with high compliance rates, resulting in sufficient samples at each time point. Furthermore, total cohort and full data subgroup analyses revealed similar results, suggesting a great robustness of our data. A limitation of this study was the use of BIA to assess body composition. BIA provides a simple, inexpensive and non-invasive alternative for dual-energy X-ray absorptiometry (DXA) measurements, and studies show high correlations between BIA and DXA [25, 26]. Nevertheless, some caution in interpreting results regarding FFM in populations with obesity is warranted, since predictive equations in conventional BIA rely on a normal hydration status. Obesity is related to variations in hydration of soft tissue (e.g. excess cellular water and greater body water in trunk region) and weight loss can also influence hydration status [27, 28]. A review showed that validation studies for use of BIA in obese populations reported an overestimation of FFM by 2.87 kg (range 1.0–5.18 kg); however, other studies found a non-significant underestimation of FFM by BIA [29]. Despite these small cross-sectional differences between BIA and reference measures, BIA will have a repeatable and constant bias when the same machine in measurement protocol is used in a longitudinal design [29]. This suggests that the restrictions of BIA are limited in our study, since we use repetitive measurements. Moreover, our large cohort also limits the influence of potential outliers. Therefore, data of the current study still provides relevant insights into the prevalence of excessive FFM loss and its determinants. Another limitation was that our cutoff values for excessive FFM loss were not specific for post-bariatric populations. However, the lack of studies on the impact of FFM loss on health parameters makes it difficult to determine how much FFM loss is harmful and therefore excessive. Nevertheless, with this study, we aimed to get some insight into the possible magnitude of this issue. Novel studies on the impact of post-bariatric FFM loss on long-term health could help to develop guidelines on FFM loss after bariatric surgery.

## Conclusion

Post-bariatric patients lost a substantial amount of FFM up to 18 months post-surgery, with highest rates of FFM loss at 3 and 6 months post-surgery. FFM loss was considered excessive in 7 to 46% of the population, dependent on follow-up moment and cutoff value. The high prevalence of excessive FFM loss emphasizes that post-bariatric care should have more focus on FFM loss and its consequences for the patient. Factors such as age, sex, preoperative BMI and type of surgery could help identifying patients at risk for excessive FFM loss. Future studies assessing dietary intake and physical activity within 6 months post-surgery are warranted to address its multifactorial etiology.

## Electronic Supplementary Material

ESM 1 Changes in fat mass (A) and fat-free mass (B) with respect to preoperative measures up to 36 months post-surgery for total cohort (red) and full data subgroup (blue). Error bars reflect standard deviation (1SD). (PNG 387 kb).

High resolution image (TIF 104323 kb).

ESM 2 (DOCX 15.7 kb).
