# Supplementary figures and images for: Rate and Determinants of Excessive Fat-Free Mass Loss After Bariatric Surgery
Source: Obes Surg. 2020 May 15;30(8):3119–26. doi: 10.1007/s11695-020-04654-6 (PMC7305251; doi:10.1007/s11695-020-04654-6)

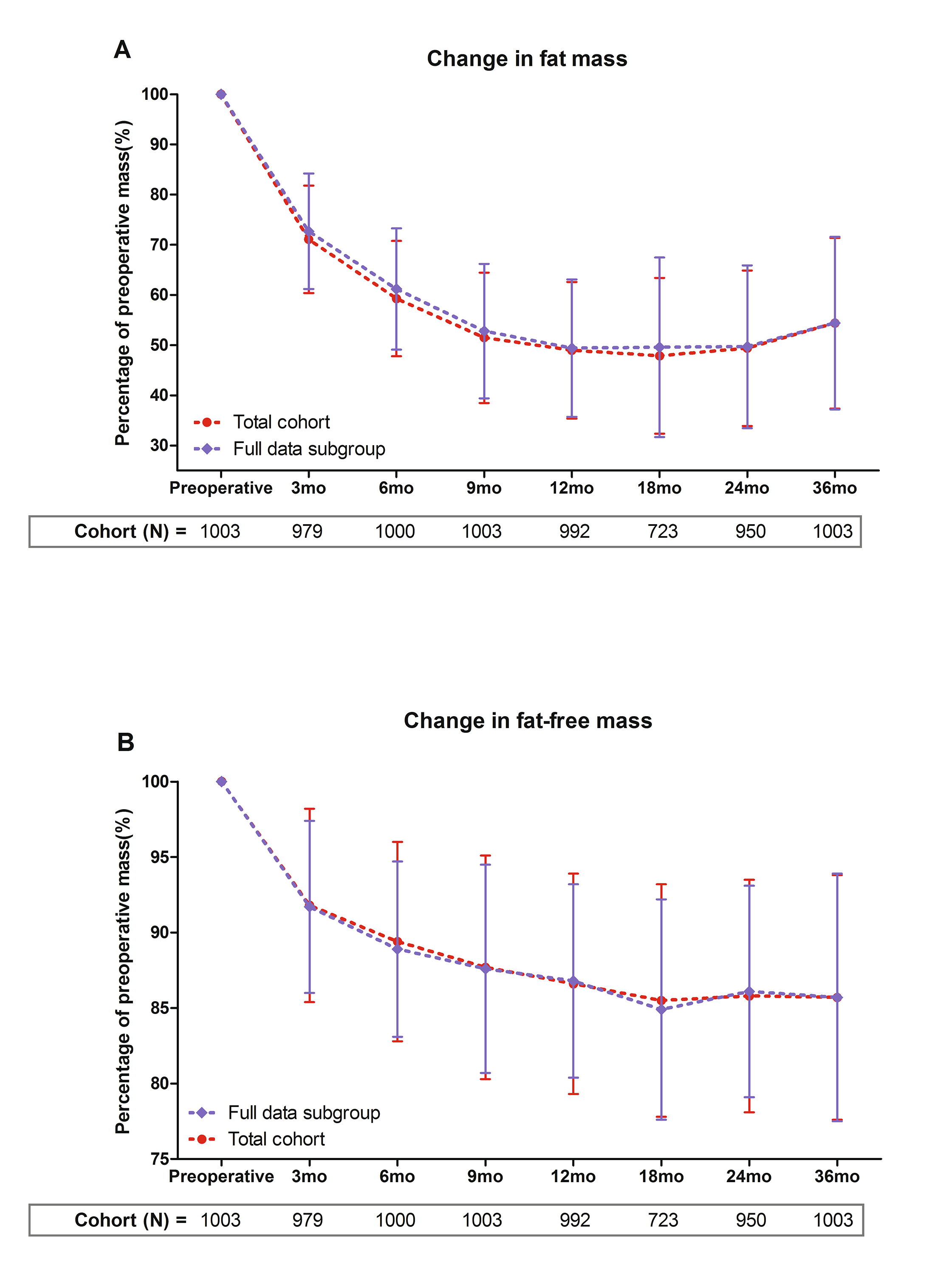

Supplement: Supplementary file 1 — Changes in fat mass (A) and fat-free mass (B) with respect to preoperative measures up to 36 months post-surgery for total cohort (red) and full data subgroup (blue). Error bars reflect standard deviation (1SD). (PNG 387 kb). [file 11695_2020_4654_Fig4_ESM.png]
