# Supplementary material for: Rate and Determinants of Excessive Fat-Free Mass Loss After Bariatric Surgery
Source: Obes Surg. 2020 May 15;30(8):3119–26. doi: 10.1007/s11695-020-04654-6 (PMC7305251; doi:10.1007/s11695-020-04654-6)
Supplement: Supplementary file 3 — (DOCX 15.7 kb). [file 11695_2020_4654_MOESM2_ESM.docx]

| **Supplemental table 1.** Logistic regression on 12-month and 24-month excessive fat-free mass loss (%FFML/WL≥25). | | | | | | | |
| --- | --- | --- | --- | --- | --- | --- | --- |
|  | **12-month** | | | **24-month** | | | |
|  | **Exp(B)** | **95% CI** | **P** | **Exp(B)** | | **95% CI** | **P** |
| *Univariate* | |  |  |  |  | |  |
| Age | 1.025 | 1.017 – 1.032 | **<.001** | 1.030 | 1.022 – 1.039 | | **<.001** |
| Sex *(ref=male)* | 0.458 | 0.377 – 0.556 | **<.001** | 0.482 | 0.389 – 0.597 | | **<.001** |
| Type of surgery *(ref=RYGB)* | 1.865 | 1.520 – 2.289 | **<.001** | 1.631 | 1.299 – 2.047 | | **<.001** |
| Preoperative BMI | 1.031 | 1.016 – 1.045 | **<.001** | 1.022 | 1.007 – 1.038 | | **.004** |
| Hypertension | 1.533 | 1.307 – 1.798 | **<.001** | 1.668 | 1.412 – 1.972 | | **<.001** |
| Dyslipidaemia | 1.482 | 1.225 – 1.792 | **<.001** | 1.767 | 1.451 – 2.152 | | **<.001** |
| Sleep apnoea | 1.550 | 1.240 – 1.938 | **<.001** | 1.555 | 1.226 – 1.971 | | **<.001** |
| Arthrosis | 1.371 | 1.089 – 1.726 | **.007** | 1.417 | 1.119-1.794 | | **.004** |
| Diabetes | 1.550 | 1.290 – 1.863 | **<.001** | 1.776 | 1.465-2.153 | | **<.001** |
| *Multivariate* | |  |  |  |  | |  |
| Age | 1.021 | 1.012 – 1.030 | **<.001** | 1.024 | 1.014 – 1.034 | | **<.001** |
| Sex *(ref=male)* | 0.519 | 0.422 – 0.639 | **<.001** | 0.547 | 0.456 – 0.722 | | **<.001** |
| Type of surgery *(ref=RYGB)* | 1.930 | 1.548 – 2.406 | **<.001** | 1.834 | 1.433 – 2.346 | | **<.001** |
| Preoperative BMI | 1.035 | 1.019 – 1.051 | **<.001** | 1.030 | 1.013 – 1.046 | | **<.001** |
| Hypertension | 1.133 | 0.934 – 1.373 | .21 | 1.137 | 0.930 – 1.389 | | .21 |
| Dyslipidaemia | 1.059 | 0.842 – 1.332 | .62 | 1.204 | 0.951 – 1.524 | | .12 |
| Sleep apnoea | 1.119 | 0.880 – 1.423 | .36 | 1.138 | 0.882 – 1.468 | | .32 |
| Arthrosis | 1.154 | 0.902 – 1.477 | .26 | 1.148 | 0.891 – 1.478 | | .29 |
| Diabetes | 1.179 | 0.945 – 1.471 | .15 | 1.310 | 1.040 – 1.651 | | **.022** |
| Overall multivariate model fit was R^2^=0.052 for 12-month and R^2^=0.058 for 24-month excessive fat-free mass loss. | | | | | | | |
